# Tranexamic acid versus oxytocin prophylaxis in reducing post-partum blood loss, in low-risk pregnant women: TRANOXY STUDY, a phase III randomized clinical trial

**DOI:** 10.1016/j.eclinm.2024.102665

**Published:** 2024-05-31

**Authors:** Antonio Ragusa, Fernando Ficarola, Amerigo Ferrari, Nicoletta Spirito, Mario Ardovino, Domenico Giraldi, Elisario Stuzziero, Denise Rinaldo, Roberto Procaccianti, Giovanni Larciprete, Caterina De Luca, Sara D'Avino, Giulia Principi, Roberto Angioli, Alessandro Svelato

**Affiliations:** a Department of Obstetrics and Gynecology, Maggiore Hospital Carlo Alberto Pizzardi Bologna, Italy; b Unit of Gynecology, Fondazione Policlinico Universitario Campus Bio-Medico, Rome, Italy; c Unit of Gynecology, Department of Surgical and Medical Sciences and Translational Medicine, Sant'Andrea Hospital, Sapienza University of Rome, Rome, Italy; d Institute of Management, MeS (Management and Health) Laboratory, Sant’Anna School of Advanced Studies, Pisa, Italy; e Department of Obstetrics and Gynecology, Ospedale Apuane, Massa Carrara, Italy; f Department of Obstetrics and Gynecology, Ospedale S.G. Moscati, Avellino, Italy; g Department of Obstetrics and Gynecology, ASST Bergamo Est, Bolognini Hospital, Seriate, Italy; h Department of Gynecology and Obstetrics, Fondazione Istituto San Raffaele G Giglio, Cefalù, Italy; i Department of Obstetrics and Gynecology, Fatebenefratelli Gemelli Hospital, Isola Tiberina, Roma, Italy; j Department of Obstetrics and Gynecology, University of Messina, Messina, Italy

**Keywords:** Tranexamic acid, Oxytocin, Postpartum hemorrhage, Hemorrhage, Obstetric labor complications

## Abstract

**Background:**

To assess the equivalence of tranexamic acid (TRAN) versus synthetic oxytocin (OXY) in reducing post-partum blood loss, in full-term patients (37–42 weeks), at low risk of post-partum hemorrhage, with vaginal childbirth.

**Methods:**

Phase III, randomized (1:1), open-label, longitudinal, multi-center, prospective clinical trial (Prot. n 63209, ClinicalTrials.gov Identifier: NCT02775773). From January 7, 2020, to June 30, 2023, a total of 256 women were enrolled at two general urban community hospitals in Italy, serving a multi-ethnic patient population with National Health Insurance. The primary outcome was to explore a potential equivalence between the two treatments (OXY and TRAN) in preventing total blood loss. Therefore, we randomized 231 women into two groups: Group A (OXY), 127 women who were administered 10UI intramuscularly within 5 min from childbirth; Group B (TRAN), 104 women to whom 1-g slow intravenous infusion was administered within 5 min from childbirth.

**Findings:**

At the time of delivery, mean blood loss for OXY group versus TRAN group was 269.12 mL versus 263.88 mL, respectively, with equivalence between the two groups. Similarly, there was equivalence in total blood loss between the OXY and the TRAN group (397.66 mL versus 405.64 mL, respectively. No statistical differences between Hb levels at admission and discharge in the two groups were reported. No difference was found in terms of additional uterotonic and surgical therapies between the two groups of patients. Neither group showed thrombotic complications at check-up performed after 7 days or after a questionnaire regarding adverse effects, subjected after 40 days.

**Interpretation:**

The study shows the equivalence of tranexamic acid versus synthetic oxytocin in post-partum blood loss prophylaxis in term patients at low risk of PPH with vaginal childbirth. The safety profiles of OXY and TRAN were similar.

**Funding:**

None.

**Research in context**

**Evidence before this study**

Preclinical data, epidemiological studies, and meta-analyses of randomized trials for PPH prevention, support the hypothesis that tranexamic acid (TRAN) in combination with oxytocin could be an effective prophylaxis for post-partum hemorrhage (PPH). Globally, several phase III studies are ongoing to assess this, though debate continues about the safety profile and efficacy of TRAN.

**Added value of this study**

Our study is the first to show that tranexamic acid is effective in preventing blood loss after delivery, even when it is used alone, and in place of synthetic oxytocin. The data show that tranexamic acid is well tolerated, acceptable to patients, and there is no evidence to suggest there is increased toxicity compared to synthetic oxytocin.

**Implications of all the available evidence**

Tranexamic acid is a low-cost generic and thermostable drug, with the potential to have a large impact on post-partum blood loss in the absence of uterine atony. We demonstrated that TRAN could be used as prophylaxis for blood loss and it is well tolerated.

## Introduction

Post-partum hemorrhage (PPH) is a serious complication that can occur both during vaginal delivery as well as during cesarean sections. Traditionally, PPH is defined as blood loss equal to or greater than 500 mL after a vaginal delivery (severe if greater than 1000 mL) and equal to or greater than 1000 mL after a caesarean section.^1^ Unfortunately, there is no unanimous consensus on the definition of PPH.^2^

The American College of Obstetricians and Gynecologists (ACOG) created a new definition: primary PPH is a cumulative blood loss of >1000 mL, independently of the route of childbirth, or blood loss accompanied by signs or symptoms of hypovolemia within 24 h after the birth process.^3^ Currently, preventive drug therapy is the most widely used method for the prevention of PPH. There are numerous and different drugs that can be used for this purpose: oxytocin, carbetocin, methylergonovine, ergometrine, prostaglandin analogs, or tranexamic acid.^4^ WHO recommend oxytocin, 10 UI, intramuscularly/intravenously, as front-line after all births for the prevention of PPH in vaginal childbirth.^5^

Tranexamic acid (TRAN) is an antifibrinolytic drug with a good safety profile. TRAN is an inhibitor of plasminogen activation, preventing the conversion of plasminogen to plasmin and, thereby, inhibiting fibrinolysis. If administered intravenously at 1 g (10 mL of a 100 mg/mL solution), TRAN shows an onset of action within 5 min and a half-life of approximately 2 h. In a meta-analysis of RCTs, the prophylactic use of TRAN after vaginal delivery, in addition to oxytocin, cord traction, and uterine massage, was found to result in a 39% reduction in the risk of PPH when compared with placeboes.^6^

TRAN has, however, at least theoretically, some advantages over oxytocin: persistence in tissues for up to 17 h post-administration, capacity to decrease maternal mortality in women with PPH, thermostability, operational advantage of overcoming logistic costs and challenges inherent to ensuring a cold chain system. TRAN, unlike OXY, has no effect on the maternal neuro-hormonal system, nor on mother-child dyad attachment. Intrapartum exposure to synthetic oxytocin, on the other hand, significantly reduces infants’ sucking capacity during skin-to-skin contact with the mother, in the first hour after birth, in a dose-dependent manner.^7^, ^8^, ^9^

For all these reasons, we decided to verify the equivalence between TRAN (EV) and OXY (IM) in the prophylaxis of postpartum blood loss in patients at term of pregnancy (37–42 weeks of amenorrhea), at low risk of PPH with vaginal childbirth. A secondary objective was to evaluate the safety profile and tolerability of TRAN compared to OXY.

## Methods

### Study design and participants

The Tranoxy Study was a prospective multi-center, randomized, unblinded, controlled trial that was conducted at two Italian general urban hospitals (Ospedale Fatebenefratelli Isola Tiberina, Rome, and Ospedale San Giuseppe Moscati, Avellino) serving a multi-ethnic patient population with National Health Insurance, as is provided in Italy. The study follows CONSORT (CONsolidated Standards of Reporting Trials) guidelines. The trial protocol was approved by the ethics Committee Toscana Nord Ovest. ClinicalTrials.gov Identifier: NCT02775773 (https://classic.clinicaltrials.gov/ct2/show/NCT02775773).

### Patients

The enrolled pregnancy population (inclusion criteria) consists of full-term patients (37–42 weeks) at low risk of PPH. Low risk of PPH refers to patients who do not report any risk factors, which therefore constitute exclusion criteria the presence of even one of these factors excluded the possibility of participating in the study: hypertension, preeclampsia, placental abruption in pregnancy, placenta previa, tocolysis 2 h before childbirth, twinning, previous PPH; obesity (BMI> 35), anaemia (Hb <7 g/dL), elective caesarean section, induction of childbirth, polyhydramnios; fever in labor, use of low molecular weight heparin. preterm pregnancy (<37 weeks), prolonged pregnancy (>42 weeks), patients with QT-Long syndrome or taking medications that can cause QT stretching, intrauterine fetal death, renal diseases, epilepsy; autoimmune diseases, presence in remote pathological anamnesis or in family history of thromboembolic events (Appendix 6 and 7).

### Randomisation and masking

Enrolled patients were randomized 1:1 and assigned to either synthetic oxytocin (OXY) (usual treatment) or tranexamic acid (TRAN) group, using a computer-generated random number of series with Excel 2016, software version 16.0. The randomization was not influenced by known risk factors for PPH. The program generated a randomization list that was reported in sealed envelopes by personnel unrelated to the study. At the time of admission, an enrollment visit was scheduled, during which a list of standardized procedures was performed (Appendix p 8). Routine checks were performed before and after treatment administration in both groups. In the OXY group (127 pt), the usual treatment with 10 UI intramuscularly was used within 5 min from childbirth. In the TRAN group (104 pt), 1 g slow intravenous (max 5 mL/min) infusion was used within 5 min from childbirth. The same treatment was applied to ensure patients’ safety in the case of postpartum hemorrhage in both treatment groups. A checkup was performed after 7 days, and a questionnaire concerning adverse effects was subjected to all the women taking part in the study after 40 days.

### Outcomes

The primary outcomes evaluated in the study are 1A) blood loss expressed in mL at the time of delivery (T0) until completion of stage III and/or completion of any vagino-perineal suture, and 1B) total blood loss computed as the sum of blood loss at the time of delivery and blood loss at 2 h after delivery (lochia). The secondary outcomes of this study were: 2A) total blood loss over 1000 mL, 2B) total blood loss over 500 mL, and 2C) Hemoglobin levels at 24 h after delivery. We chose both the cutoff of 1000 mL and the cutoff of 500 mL since a blood loss equal to or greater than 500 mL after a vaginal delivery is traditionally defined as PPH, but when equal to or greater than 1000 mL is defined as severe PPH. The safety outcome was prespecified; all other safety outcomes were based on adverse-event reporting.

### Statistics

The sample size of the study was determined based on the primary efficacy outcome of blood loss at the time of delivery (T0). A review of the literature for the calculation of sample size showed that the average blood loss at the time of delivery after administration of IM OXY is equal to 300 mL. Therefore, bearing in mind that the objective of the study is to demonstrate the equivalence of TRAN (EV) versus OXY (IM) in reducing bleeding after delivery, taking a Δ = 150 mL (Δ = maximum difference between treatments not clinically relevant) to demonstrate equivalence between the two treatments, 87 subjects were needed for each treatment sector. The value Δ = 150 mL was chosen because a difference of 150 mL is not clinically relevant; In fact, a value of 450 mL (300 mL + 150 mL) is still lower than the critical value of 500 mL which defines PPH. The sample size was determined by considering a study power of 90%, a standard deviation of the overall population of 300, with a 1:1 randomization for each comparison and a two-tailed α error of 5% (Appendix p 14). Other statistical considerations can be found in the Appendix (p 10).

We described the baseline characteristics of our study population and of the two groups (OXY versus. TRAN) separately. We performed the Mann–Whitney U-test for continuous variables, and the chi-square test for categorical variables to compare the outcome variables between the two study groups.

We then performed an equivalence test using the ‘two one-sided t-tests’ (TOST) procedure, setting the parameter Δ = 150 mL. To satisfy the assumption of normality of the distribution of the data, we expressed the primary outcome variables “T0 blood loss” and “total blood loss” in logarithmic form and checked the normality of the distribution by the Shapiro–Wilk test.

We reported categorical variables as counts (and percentages) and continuous variables as median (and interquartile range) or mean (and standard deviation). Statistical significance was set at a p-value <0.05. All analyses were performed on Stata Software version 17.0 (Stata-Corp, LLC, College Station, Texas, USA).

### Ethics

The trial protocol was approved by Ethics Committee for Clinical Trials of the Region of Tuscany. Section: VASTA NORTHWEST AREA. Ethics Committee Register Number: 2016-1129. Prot. n 63209.

The trial was performed following the principles of the Declaration of Helsinki. The authors assume responsibility for the accuracy and completeness of the data and analyses, as well as for the fidelity of the trial and this report to the protocol. Informed consent was obtained from all individual participants included in the study.

### Role of the funding source

This research received no specific grant from any funding agency in the public, commercial, or not-for-profit sectors.

## Results

### Patients’ characteristics

**Fig. 1:**
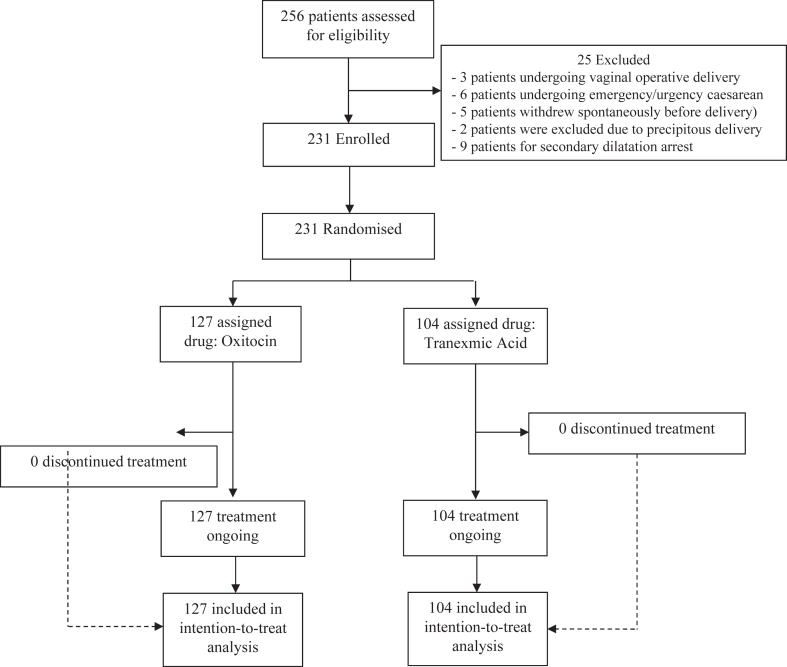
**Study flowchart**. *Legend*. This flowchart reports the study design. It shows the number of patients eligible for the study, from which patients who had no prespecified exclusion criteria were recruited. The enrolled patients were randomized into two groups: standard oxytocin treatment and tranexamic acid treatment. As the treatment was immediate and single administration after delivery, no patients discontinued the study, and all enrolled patients were included in the data analysis.

From January 7, 2020, to June 30, 2023, a total of 256 women were enrolled at two centers, and after the exclusion of 25 women, a total of 231 were randomized, 127 in the OXY group and 104 in the TRAN group respectively (Fig. 1). Overall, the two groups were balanced with respect to baseline characteristics, as shown in Table 1. The median age was similar in the two groups (32 years), while the median gestational age was slightly lower in the TRAN group (39 versus 40 weeks). Women in the TRAN group were less likely multiparous but had a slightly higher BMI (24.6 versus 25.0) and baseline Hemoglobin levels (11.9 versus 11.7 g/dL).

**Table 1:** Study population baseline characteristic.

Population baseline characteristics	Study population (n = 231)	OXY group (n = 127)	TRAN group (n = 104)
Age, median (IQR)	32.0 (29.0, 36.0)	32.0 (29.0, 36.0)	32.0 (29.0, 36.0)
Gestational age, median (IQR)	39.0 (39.0, 40.0)	40.0 (39.0, 40.0)	39.0 (39.0, 40.0)
Days of pregnancy, median (IQR)	279.0 (273.0, 282.0)	280.0 (275.0, 283.0)	277.0 (273.0, 281.0)
Previous deliveries, median (IQR)	1.0 (0.0, 1.0)	1.0 (0.0, 1.0)	0.5 (0.0, 1.0)
BMI, median (IQR)	24.9 (22.5, 27.3)	24.6 (22.4, 26.8)	25.0 (22.6, 27.6)
Baseline Hb, median (IQR)	11.8 (11.0, 12.6)	11.7 (11.0, 12.5)	11.9 (11.0, 12.6)

### Follow-up

All randomized patients received the prophylaxis: OXY 10 UI intramuscularly within 5 min after childbirth (Group A); TRAN 1 gr slow intravenous (max 5 mL/min) infusion within 5 min after childbirth (Group B).

25 patients were excluded from the study for different reasons:

- 2 patients due to precipitous delivery (dilation rate greater than 5 cm/h).
- 9 patients for active phase protraction and arrest (once 6 cm cervical dilation is achieved); no cervical dilation after 4 h of adequate contractions, with ruptured membranes or no cervical dilation after 6 h of inadequate contractions, with ruptured membranes, and despite oxytocin administration. 4/9 patients gave birth vaginally; 5/9 patients gave birth by caesarean section.
- 3 patients undergoing vaginal operative delivery using an omnicup suction cup.
- 6 patients undergoing emergency/urgency caesarean section for alterations in the cardiotocographic trace.
- 5 patients withdrew spontaneously before delivery for personal reasons, in accordance with the provisions of the informed consent signed by them (Fig. 1).

### Outcomes

**Fig. 2:**
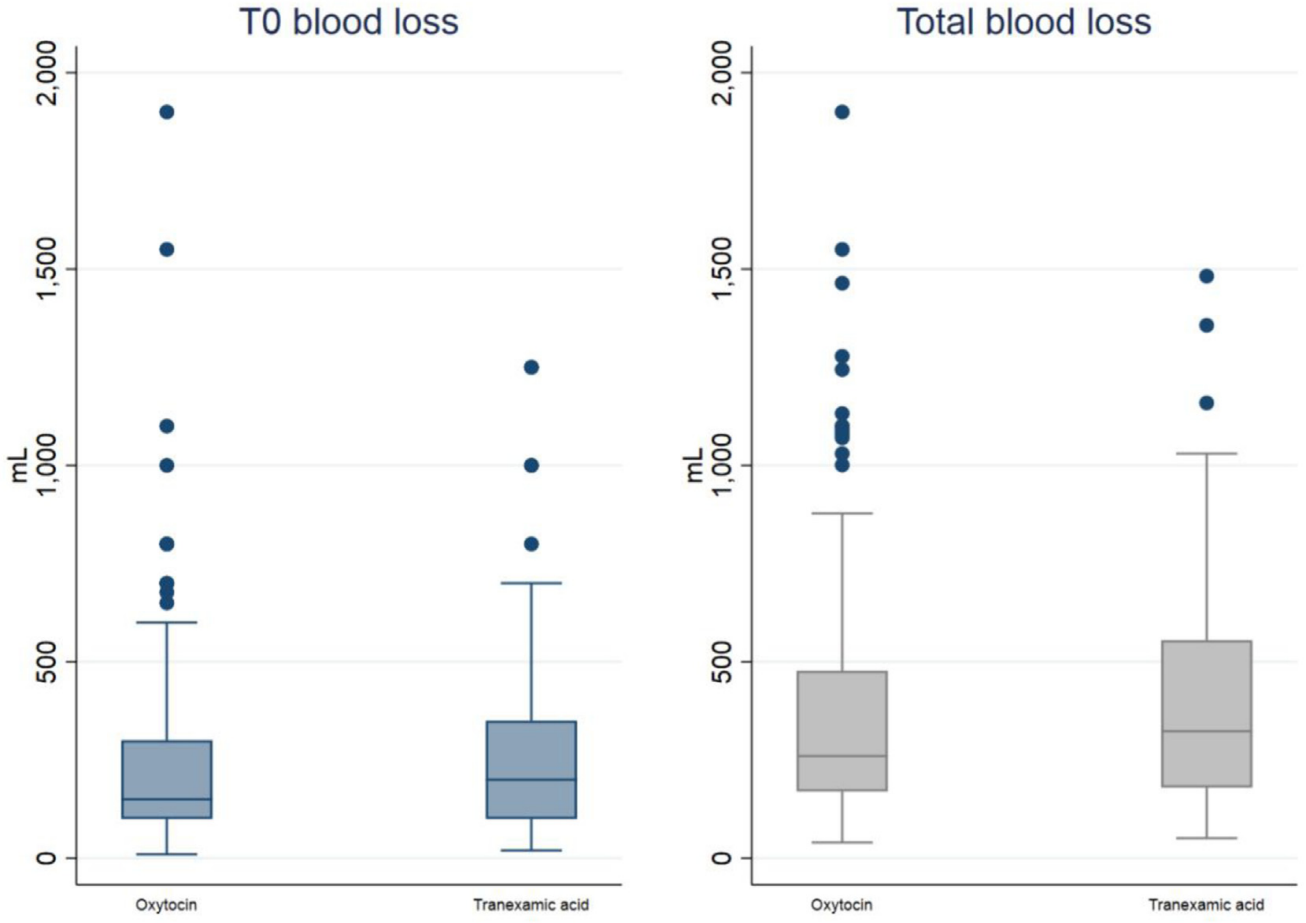
**Boxplots for the two primary outcomes**. *Legend*. In boxplots, the box ranges from a quartile in the lower range (25th percentile of data) to a quartile in the upper range (75th percentile). It also contains a line in the median value (50th percentile). Whiskers plots range from the quartile with a low value and a low adjacent value to the quartile in the upper range and upper adjacent value. So, we have: Lower Adjacent Value = lower quartile—3/2 IQR (interquartile range); and Upper Adjacent Value = upper quartile + 3/2 IQR. Observations after the lower and upper adjacent value are plotted in terms of points and represent outside values.

**Fig. 3:**
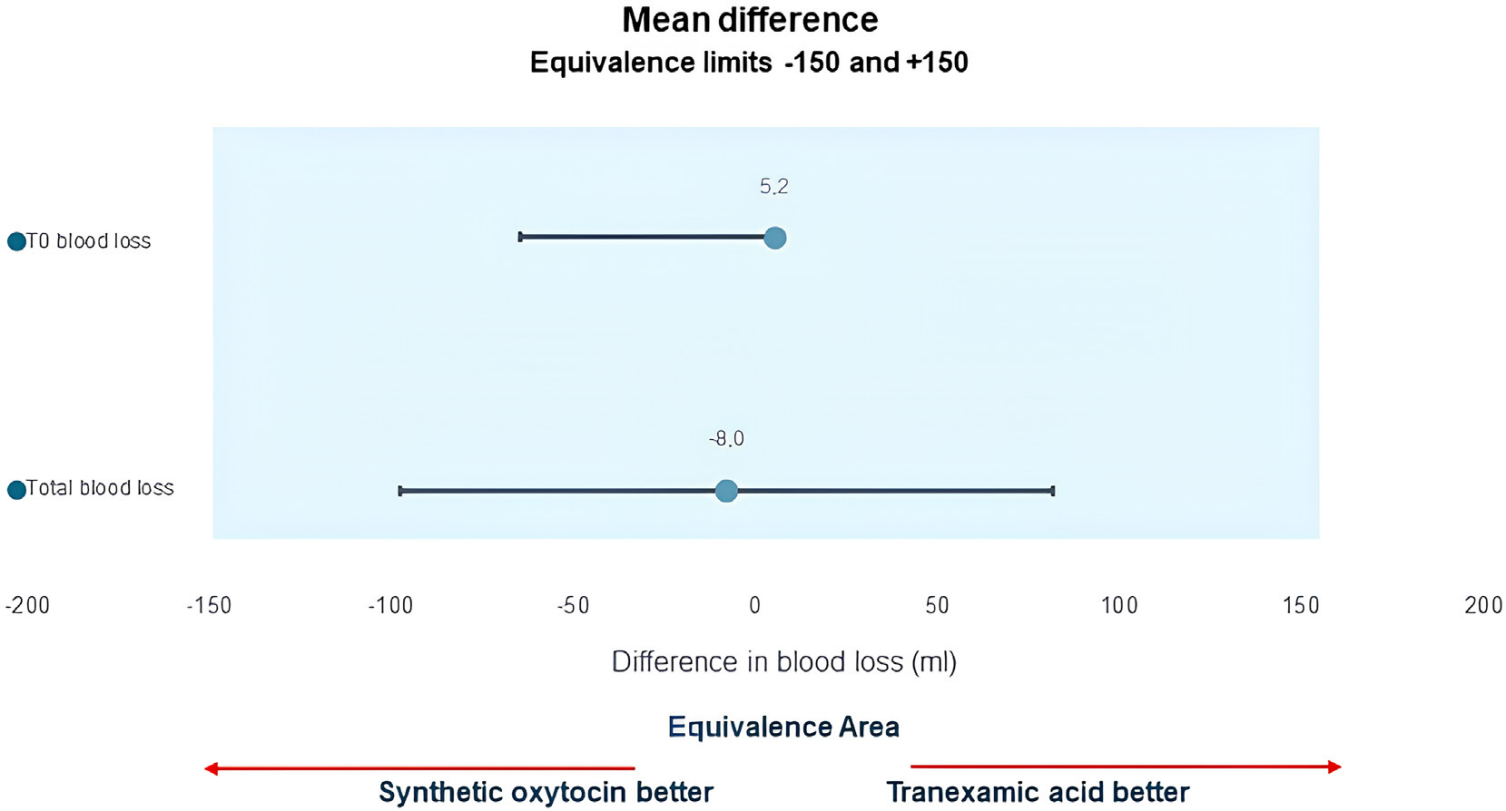
**TOST procedure for equivalence between the groups**. Mean differences in T0 blood loss and total blood loss between the two treatments (synthetic oxytocin—tranexamic acid) and corresponding 95% confidence interval estimated by means of the TOST procedure. The light-blue area represents the area of equivalence bounded by two vertical dashed lines indicating the equivalence limits (from −150 mL to +150 mL).

The mean blood loss at T0 (time of delivery) was 269.12 mL (95% CI: 219.39–318.84) in the OXY group, and 263.88 mL (95% CI: 215.25–312.52) in the TRAN group (Table 2). Given an equivalence margin of 150 mL (5.01 in logarithmic units), which represents a non-clinically relevant difference in blood loss after delivery, the TOST procedure revealed that the two treatments were equivalent (Table 2, Figs. 2 and 3).

**Table 2:** Equivalence test for primary outcomes.

	T0 blood loss		log (T0 blood loss)		TOST
	Mean	95% CI	Mean	95% CI	
Synthetic oxytocin (n = 127)	269.12	219.39–318.84	5.19	5.03–5.35	Test for difference
Tranexamic acid (n = 104)	263.88	215.25–312.52	5.19	5.02–5.37	H0: no difference (diff = 0)
					p-value >0.05 (not rejected)
Overall (n = 231)	266.76	231.99–301.53	5.19	5.07–5.31	
					Test for equivalence
Difference	5.23	−64.81 to 75.28	0.00	−0.24 to 0.24	H0: no equivalence (|θ| ≥ Δ)
					p-value <0.05 (rejected)
p-value for normality	<0.001 (not normal)		>0.05 (normal)		
Equivalence limits (Δ)	150 mL		5.01 (log units)		Conclusion: equivalence


	T0 blood loss		log (T0 blood loss)		TOST
	Mean	95% CI	Mean	95% CI	
Synthetic oxytocin (n = 119)	397.66	334.36–460.96	5.68	5.54–5.82	Test for difference
Tranexamic acid (n = 91)	405.64	344.23–467.04	5.75	5.59–5.90	H0: no difference (diff = 0)
					p-value >0.05 (not rejected)
Overall (n = 210)	401.12	356.81–445.43	5.71	5.61–5.81	
					Test for equivalence
Difference	−7.97	−97.61 to 81.66	−0.07	−0.27 to 0.14	H0: no equivalence (|θ| ≥ Δ)
					p-value <0.05 (rejected)
p-value for normality	<0.001 (not normal)		>0.05 (normal)		
Equivalence limits (Δ)	150 mL		5.01 (log units)		Conclusion: equivalence

The mean total blood loss in the OXY and TRAN groups was 397.66 mL (95% CI: 334.36–460.96) and 405.64 mL (95% CI: 344.23–467.04), respectively (Table 2). The TOST procedure showed between-group equivalence within a margin of 150 mL (5.01 in logarithmic units) (Table 2, Figs. 2 and 3).

As for the secondary outcomes in Supplementary Table S2 (Appendix p 12 and 13), we found no significant difference between the OXY and the TRAN groups in the proportion of blood loss over 1000 mL (16.5% versus. 16.3%, respectively) and in the proportion of blood loss over 500 mL (28.3% versus. 39.4%, respectively), but, at the same time, we were not able to demonstrate equivalence by means of the TOST approach. On the contrary, the TOST procedure showed equivalence in the Hemoglobin levels at 24 h after delivery (mean levels of 10.6 g/dL in both groups).

In this study, all cases of PPH were treated according to Italian national guidelines.^10^

The need to use drugs, invasive maneuvers such as intrauterine devices, or surgical procedures in order to treat postpartum bleeding, was necessary in only one case, belonging to the OXY group, after the administration of 1 g of tranexamic acid IV and 2 ampoules of methylergometrine IM, resorting intrauterine hemostatic balloon (Bakri Balloon).

Surgical procedures were not necessary in either group to treat and resolve postpartum bleeding. No adverse event was reporting. No thrombotic complications or significant hemodynamic changes were recorded in both groups. All safety outcomes are reported in Supplementary Table S3 (Appendix p 13). Check-up performed after 7 days, and a follow-up after 40 days showed no differences in adverse event incidence between the two treatment groups.

## Discussion

This study demonstrates, for the first time, that tranexamic acid, administered alone and for slow intravenous infusion within 5 min of vaginal childbirth, is equivalent to intramuscular oxytocin in preventing postpartum blood loss in term patients at low risk of PPH. Thus, suggesting its potential use for PPH prophylaxis in the absence of uterine atony.

PPH is one of the leading causes of maternal mortality and morbidity worldwide.^11^ Available evidence supports the use of tranexamic acid associated with uterotonic to reduce PPH in women of all risk categories in both industrialized and developing countries.^6^ There is evidence demonstrating the usefulness of TRAN in the treatment of PPH when administered within 3 h after birth in combination with oxytocin.^12^

Current knowledge indicates that fibrinogenemia levels are higher in pregnant women than in non-pregnant women. Fibrinogenemia levels increase significantly in the third trimester with estrogen levels and decrease during the third stage of labor (delivery of placenta and membranes). This decrease in fibrinogenemia is due to intravascular fibrin deposition during postpartum, which leads to an increase in fibrinogen consumption.^10^^,^^13^ In fact, tissue biopsies studies of the placental bed using an electron microscope reveal that, immediately after a normal childbirth, an extravascular fibrin network forms on the endometrial surface.^14^ These physiological mechanisms have led us to hypothesize that TRAN, as an antifibrinolytic agent, may be an effective prophylaxis in the control of postpartum bleeding.

The therapeutic inhibition of fibrinolysis has already proven effective in other clinical settings, such as cardiac surgery, hepatic, traumatology, and neurosurgery, reducing the risk of blood transfusions, the average volume of blood transfused, the need for reoperation, without increasing the thromboembolic risk^15^, ^16^, ^17^ Clinical Randomization of an Antifibrinolytic in significant hemorrhage has shown that administration of TRAN (1 g loading dose in 10 min followed by 1 g in 8 h) within 3 h of trauma, reduces all-cause mortality by 16%–14.5% within four weeks (hemorrhage, vascular occlusion, myocardial infarction, stroke, pulmonary embolism) in the traumatized patient, without increasing thrombotic events (pulmonary embolism, deep vein thrombosis).^18^ The survival benefit is only evident if TRAN treatment is initiated within 3 h of injury and does not increase the risk of cardio-vascular occlusive events.

Despite this, not all results agree: in fact, a 2022 RCT by the Maternal-Fetal Medicine Units Network demonstrated that prophylactic administration of TRAN, during cesarean delivery, did not reduce the need for packed RBC transfusion, but did, however, modestly decrease the need for uterotonics.^19^ Nevertheless, numerous studies have evaluated the efficacy of TRAN, in addition to the administration of OXY, in the prevention of PPH after cesarean delivery.^20^^,^^21^ In all these studies, except for Sentürk MB et al., C-sections were elective and not urgent.^20^^,^^21^ These studies all reported significantly less mean postpartum blood loss in women who received TRAN, and no detectable adverse effects on their vital signs (blood pressure, heart rate, and respiratory rate) or thrombosis.^20^^,^^21^ Prophylactic intravenous TRAN before cesarean sections has recently proven to be helpful in preventing perioperative bleeding in women.^22^

Unfortunately, only a few studies have evaluated the effectiveness of TRAN for the prevention of PPH after vaginal delivery, and none of them evaluated tranexamic acid as the only therapeutic agent as we did in this study.^23^^,^^24^ Women who received prophylactic tranexamic acid after vaginal delivery had a significantly lower incidence of primary PPH and lower mean blood loss mean difference. The risk of thrombotic events was not increased in the tranexamic acid group.^6^

There are numerous studies that have evaluated the effectiveness of tranexamic acid in the prophylaxis of PPH, but none have evaluated its use alone, nor in combination.^13^^,^^25^ We soon hope to know more about TRAN prophylaxis and vaginal childbirth, as WOMAN-2 Trial, designed to assess if TRAN prevents PPH in women with moderate to severe anemia undergoing vaginal delivery, is currently recruiting participants (ClinicalTrials.gov Identifier: NCT03475342), But for now, the evidence in favor of using TRAN after vaginal delivery is still unclear; this is why we designed and conducted this study.

Furthermore, considerable advantages of TRAN over OXY may be hypothesized. First, TRAN is stable over a wide temperature range, from −20 °C to +50 °C for more than 12 weeks.^26^ Therefore, it could be used in countries such as sub-Saharan Africa, where the availability of this drug to the women who have recently given birth, could reduce maternal mortality from PPH by 30% (about 22,000 deaths per year).^27^ Second, a further advantage of TRAN is that it has no neuroendocrine effects on neonatal maternal dyad attachment.^28^ In addition, other studies indicate that exposure to synthetic oxytocin during delivery is an independent risk factor for a delay in gross- and fine motor development in infants.^29^

A possible advantage that may result from the use of TRAN in the prevention of PPH (in the absence of uterine atony) is that it does not contraindicate the subsequent use of uterotonics, which remains contemplated and recommended in the event of PPH (which may occur despite prophylaxis, whether with tranexamic acid or synthetic oxytocin). It is indeed possible that, in cases where oxytocin has to be administered after previous prophylaxis with tranexamic acid, oxytocin receptors are more sensitive to the molecule, resulting in lower receptor saturation. However, further studies are needed to prove these hypotheses.

The main limitation of this study is due to the unblinded study design, which does not allow for smoothing out the selection bias in the different centers participating in the study. In fact, the absence of double-blind was due to the two different routes of administration: one intramuscular and the other intravenous, it was not possible to hide the randomly administered and chosen drug from the delivery room operators. It would be very interesting to repeat the study, using intravenous oxytocin and comparing it with intravenous tranexamic acid, to see if again, the two drugs are equivalent in preventing postpartum hemorrhage.

Another limitation is the lack of pharmaco-economic evaluation, which, however, was not an objective of our study, but can be evaluated in subsequent studies. However, an interesting article published by Li et al. showed that the routine use of TRAN as an early treatment for PPH is highly cost-effective in Nigeria and Pakistan, and is likely to be cost-effective in countries in sub-Saharan Africa and southern Asia with a similar baseline risk of death due to bleeding.^30^ Although this is the first analysis to assess the cost-effectiveness of TRAN for the treatment of PPH, their results are largely consistent with those of previous economic evaluations that have demonstrated the cost-effectiveness of TRAN for the treatment of excessive blood loss in other patient groups, including the treatment of patients with hemorrhagic trauma and those undergoing elective surgery.^31^, ^32^, ^33^ If the additional costs for PPH treatment with intravenous TRAN are acceptable for low-middle income countries, this will translate into an equally affordable, if not lower, cost for PPH prevention. In fact, prophylactic treatment has a lower cost than emergency treatment in terms of instrumentation, drugs and personnel required.

Other limitations are the absence of data regarding breastfeeding after delivery which is a strong stimulator of oxytocic secretion and the discrepancy between the sample size of the two study arms probably due to the need to obtain two homogeneous groups. Finally, since the sample size was calculated on the primary outcome, the study was underpowered to demonstrate equivalence (although there was no statistically significant difference) between the two groups in the percentage of blood loss above 500 mL and above 1000 mL.

In conclusion, our trial suggests the equivalence of tranexamic acid versus synthetic oxytocin in post-partum blood loss prophylaxis in term patients at low risk of PPH undergoing vaginal childbirth. Potentially suggesting the systematic use of tranexamic acid instead of oxytocin in PPH prophylaxis in case there is no clear evidence of uterine atony during childbirth. Also, safety profiles of OXY and TRAN were similar. Differently from the previous literature where PPH prophylaxis has been carried out with fibrinolytic in addition to uterotonic drugs, our study is the first that has set the objective of demonstrating the equivalence between the two drugs and, therefore, the possibility of substituting OXY with TRAN alone. Thus, this study should be interpreted as a pioneering study that can serve as a basis for designing further double-blinded studies to confirm our results.

## Contributors

**All authors read and approved the final version of the manuscript.** All authors wrote, reviewed, and revised the original manuscript. AR, NS, ES, AS conceptualized the study. FF, AF did the formal analysis. AR, FF, NS, ES, AS contributed to the methods. AR, ES, RA, AS supervised the study. AR, FF, MA, DG, DR, RP, GL, CDL, SD, GP coordinated the trial and recruited participants. AR, FF, AF, AS curated the data. All authors (AR, FF, AF, NS, MA, DG, ES, DR, RP, GL, CDL, SD, GP, RA, AS) had full access to all the underlying data in the study and accepted responsibility to submit for publication. All authors have accessed and verified all the data in the study.

## Data sharing statement

Requests for data should be directed to the corresponding author (FF). Data requests from researchers who provide a methodologically sound proposal will be considered up to 36 months from the date of publication. The request must include details about the research hypothesis, the variables required, and the statistical methodology that will be employed.

## Declaration of interests

We declare no competing interests.

## Appendix A Supplementary data

Protocol Tranoxy
