# Supplementary material for: Tranexamic acid versus oxytocin prophylaxis in reducing post-partum blood loss, in low-risk pregnant women: TRANOXY STUDY, a phase III randomized clinical trial
Source: eClinicalMedicine. 2024 May 31;73:102665. doi: 10.1016/j.eclinm.2024.102665 (PMC11169955; doi:10.1016/j.eclinm.2024.102665)
Supplement: Protocol Tranoxy [file mmc1.pdf]

Equivalence of tranexamic acid vs oxytocin prophylaxis in reducing post-partum blood loss, in low-risk pregnant women: TRANOXY STUDY, a randomized clinical trial.

Antonio Ragusa et al.

PROTOCOL

SYNOPSIS

Vers. 4.0 del 23/01/2017

**Title**	Longitudinal clinical, controlled, randomized, open-label, phase III study to assess the equivalence of tranexamic acid (TXA) vs oxytocin (OXY) in reducing post partum haemorrhage (PPH) in patients at the end of pregnancy (37-42 w), at low risk of PPH.
**Study ID**	TRANOXI 2016
**Codice EudraCT**	2016-002047-42
**ClinicalTrial.Gov**	NCT02775773
**Promoter of the study**	Azienda USL Toscana Nord Ovest- Unit of Obstetrics and Gynecology<br>Hospital “Apuane”-Massa
**Principal Investigator**<br>coordinator,enrolment/randomization, dosage IMP	Azienda USL Toscana Nord Ovest<br>Hospital “Apuane”-Massa<br>Unit of Obstetrics and Gynecology<br>Dr. Antonio Ragusa
**Co-investigators**:<br>enrolment/randomization, IMP dosage, possession of drugs register, compilation eCRF	Hospital “Apuane”-Massa<br>Unit of Obstetrics and Gynecology-<br>Dr.ssa Alessandra Kemeny<br>Dr. Alessandro Svelato<br>Dr.ssa Lorenza Marconi
**Co-investigators**:<br>administration of questionnaires for ancillary study	Hospital “Apuane”-Massa<br>Unit of Pediatris - Dr. Armando Giovannoni<br>Unit of Psicology- Dr. A. Del Rosso
**Co-investigators**:<br>enrolment/randomization, IMP dosage	Hospital “Apuane”-Massa<br>Nurses and midwives operating in Unit of Obstetrics and Gynecology
Version and date of the protocol	**vers. 4.0 del 23/01/2017**

Background

PPH means a blood loss equal to or greater than 500 ml after a vaginal delivery (the bleeding is defined severe if it exceeds 1,000 mL) in 24 hours. PPH is called “primary” when blood loss arises within 24 hours after birth. PPH is one of the principal causes of mortality and morbidity in the obstetric population, causing around a quarter of maternal deaths per year and represents the leading cause of maternal death in the world *(1)*. Most deaths occur within 24-48 hours after delivery *(2);* 66% of deaths caused by PPH are attributable to " substandard care". *(3).*
PPH is also responsible for 73% of all serious morbidity during pregnancy and is the leading cause of access to obstetric intensive care units.*(4)*The ability to prevent this disease must therefore be considered a clinical priority. The causes of PPH are briefly summarized in the four **T**: **T**one in relation to possible anomalies of uterine contraction; **T**issue for the retention of placental or amniocoriale tissue; **T**rauma es. uterine rupture, cervical laceration, uterine inversion or lacerations of the birth channel; **T**hrombin in relation to blood coagulation disorders.
The cornerstone of the treatment of PPH are: the maintenance of uterine contractility obtained by physical and/or pharmacological means; the maintenance or support of circulation with proper hydration; the prevention or treatment of hemorrhagic coagulopathy.*(5,6)*
Active treatment of the third stage of labor reduces the prevalence of PPH by about 60%*(7)*.
The active treatment of the third stage of labour is made up of three elements:
• early clamping of the umbilical cord
• Controlled cord traction

• dosing of uterotonic before the third stage of labour.<br>Of these three interventions, only the latter continues to have a role of unanimously recognized efficacy. Currently, the only really important intervention to reduce the prevalence of PPH is the administration of 10 IU OXY before the third stage of labor.<br>_(8)_For women with no risk factors for PPH, who deliver vaginally, intramuscular OXY (10IU) is therefore the prophylactic treatment of choice in the third stage of labour.<br><br>Oxytocin bolus may be inappropriate for women with major cardiovascular disorders, in these patients it is extremely important to evaluate a therapy that does not involve the cardiovascular system. TXA could be the proper medication._(6)_<br>A randomized, multicenter trial on 144 patients in 2011 showed that high doses of TXA (4 g) significantly reduces the extent of blood loss in patients with PPH (> 800 ml of loss after vaginal delivery), the duration of the loss, transfusion requirements and the need for invasive procedures, in the absence of side effects, except for two cases of thrombosis in the TXA group [9]<br>Alterations of haemostatic mechanisms in PPH are poorly understood and the role of coagulation in labour was recently revalued after the results of a prospective study conducted in France from 2002 to 2004. The study was designed to assess if changes in coagulation factors are predictive of the severity of the PPH. This study showed that there is a possible link between the reduction of fibrinogen and outcomes (PPH), with a risk of severe bleeding increased by 2.63 (95% CI) times every 1g / L decrease in fibrinogen levels. However, the study did not provide any explanation of the causes of fibrinogen decrease, in other words, at this time, it is not possible to know whether the reduction of fibrinogen is a cause or a consequence of PPH_(10)._<br>Fibrinogen levels are higher in pregnant women than in non–pregnant ones, and they increase considerably in the third quarter of pregnancy in relation to estrogen levels.<br>_(11)_. In post-partum, following the third stage of labor, there is a drop in estrogen levels, that leads to a reduction of plasma fibrinogen, which may persist in the early post-partum period.<br>Due to this mechanism, we can suppose that TXA, as an antifibrinolytic agent, may be an effective therapy against the control of post-partum bleeding._(9)_<br>However, PPH recognizes various etiologies; hyperfibrinolysis, on which the action of the drug is focused, is not always present and there is the possibility that TXA is not effective in the case of hypofibrinolysis, which might explain why, in some studies, the administration of TXA did not result in significant reductions in blood loss._(10)._<br>CRASH 2 study has shown that high doses of TXA (1 g dose of load in 10 minutes followed by 1 g in 8 hours) reduces mortality (from 16% to 14.5 %) for all the causes in the traumatized patient , without increase of thrombotic events (pulmonary embolism , deep vein thrombosis)_(12)._<br>However, the therapeutic inhibition of fibrinolysis has proved effective in reducing bleeding in various clinical situations associated with the activation and dysregulation of the fibrinolytic system, including cardiac, liver, trauma and neurological surgery, and also obstetric hemorrhages.<br>The results of a randomized controlled double-blind vs. placebo study, have demonstrated the effectiveness of TXA in reducing bleeding during and after cesarean delivery_(13)_. Another randomized, double-blind vs. placebo study conducted on 454 patients showed that the addition to the active treatment of the third stage of labor (OXY10 IU within two minutes of birth, early cord clamping, controlled traction) of 1g of tranexamic acid intravenously within five minutes from disengagement of the shoulder, reduces the amount of blood loss in a vaginal delivery, in fact, the frequency of PPH was lower in the experimental group [1.8 %] compared to controls [6.8%].<br>This advantage was not seen for losses greater than 1000 mL. All this without the occurrence of thrombotic events_(14)_. Finally, as suggested in a recent review, as the TXA is heat-stable and preservable for a long time without the use of equipment, it could be the ideal drug, in particular for sub-Saharan Africa, where the majority of births take places at home or in clinics with limited technical resources and no electrical power_(15)._

**Objective**	
**Primary objective**	1. to assess that TXA (ev) is equivalent to OXY (im) in reducing blood loss in the post-partum period (ml) in patients at the

	end of pregnancy (37-42 w) at low risk of PPH.
**Secondary objectives**	xxxxxxxxxxxxxxxxxxxxxxxxxxxxxxxxxxxxxxxxxx<br><br>To assess if the treatment with TXA (ev) reduces:<br><br>1. the number of hemodynamic changes: hypotension (number of women with PA < 100/60 mm/Hg) and increased heart rate (number of women with FC > 60 bpm) than OXY in the study population.<br><br>2. the use of additional drugs (Oxytocin, Sulprostone, Misoprostol, Methylergometrina, tranexamic acid, seven factor) for the treatment of PPH compared to OXY.<br><br>3. the need for surgical maneuvers for bleeding control (need of intrauterine balloon or uterine compression sutures for surgical treatment of the PPH or embolization or hysterectomy).<br><br>4. numbers of patients affected by anemia (Hb < 7 g / dL) in the second days from delivery and the need for blood transfusions.<br><br>5. nausea between delivery and discharge<br><br>6. vomiting between delivery and discharge<br><br>7. headache between delivery and discharge<br><br>8. dyspnoea between delivery and discharge<br><br>9. endometritis after delivery
**Safety objectives**	xxxxxxxxxxxxxxxxxxxxxxxxxxxxxxxxxxxxxxxxxx<br><br>The following will be analyzed:<br><br>- thromboembolic events: deep vein thrombosis, pulmonary embolism, cerebral embolism, arterial thrombosis.<br>- Hematuria/obstuction of the urethra<br>- convulsions<br>- visual disturbances (altered color perception)<br>- adverse events, both serious and not serious, not indicated in the RCP

**Study design**	Longitudinal clinical, controlled, randomized, open-label, phase III study. The study includes three treatment groups:<br>• Group A: (IMP 1 Test) : TXA 500 mg/2 vials (=1 gram) slow intravenous (max 5ml/min) infusion within 5 minutes from delivery: (before the third stage of labor)<br>• Group B (IMP2 comparator): OXY 5 UI/ml 2 vials (10 Units) intramuscularly within 5 minutes from delivery (before the third stage of labor)<br>The study provides a hypothesis of equivalence between the two treatments, therefore will be tested the following hypotheses of equivalence: group A vs Group B.
**Population**	Patients at the end of pregnancy (37-42 w) at low risk of PPH age major or equal to 18 years old.<br>Mean by low-risk patients without any of the following risk factors: hypertension/preeclampsia, placental abruption during pregnancy, placenta previa, tocolysis two hours before giving birth, twin pregnancy, previous PPH, obesity (BMI > 35), anemia (Hb < 7g/dL), elective caesarean section, induction of labor, retention of placental material, polyhydramnios, fever during labor, use of heparin low molecular weight<br>Eligibility will be verified at the admission visit (V0),according to the inclusion criteria, and after signing the informed consent.
**Number of subjects to be enrolled**	The Sample Size has been determined using the principal efficacy outcome (blood loss in two hours after delivery). A review of the literature showed that blood loss 2 hours after birth and after administration of oxytocin im is 300ml; Therefore, if the final aim of the study is to demonstrate the equivalence of TXA (ev) vs. OXY (im) in reducing bleeding loss 2 hours after delivery, assuming a Δ=150ml (Δ = Maximum Difference Between Treatments not clinically significant), to demonstrate the equivalence between the two treatments, 128 subjects are needed for each treatment study. We have considered the value Δ = 150ml because a difference of 150 ml is not clinically relevant; a value of 450 ml (300 ml + 150 ml) is less than

	the critical value of 500 ml. The sample size has been determined considering a power = 80%, standard deviation = 160, a randomization 1:1 to each comparison, an α two-tail error = 5%
**Treatment**	The study involves the dosing of TXA and OXY according to the following treatment scheme<br>1. Group A: (IMP 1 Test): TXA 500 mg /2 vials (=1 gram) slow intravenous (max 5ml/min) infusion within 5 minutes from delivery: (before the third stage of labor)<br>2. Group B (comparison): OXY 5 UI/ml 2 vials (10 Units) intramuscularly within 5 minutes from delivery (before the third stage of labor)<br>The clamping of the umbilical cord will be executed within 30/60 seconds after birth. The controlled traction of the umbilical cord will not be executed
**Method of randomization**	Patients who meet all the eligibility requirements will be randomized (1:1).Will be used system blocks to balance the asymmetry numerical of patients assigned to three treatment studies. This system will allow a similar number of patients even was stopped early the trial.<br>The randomization codes will be generated by the computer system of the web randomization (procedures of randomization).<br>256 subjects will be randomized .
**Outcomes**	
**Primary outcome**	1. blood loss measurement expressed in ml<br>- immediately after delivery (until the end of III stage of labor and/or vagino-perineal suture)<br>- two hours after delivery<br>The measurement of the overall blood loss at delivery (in ml) will be performed by the graduated bag, placed under the pelvis of the patient immediately after delivery. The measurement two hours after childbirth will be performed by weighing the absorbent material, (number of grams equals number of milliliters of blood - dry weight of the absorbent material). The overall loss of blood in mL two hours after delivery will be performed by adding the two collections.<br>-<br>- 2. evaluation of the number of cases with global blood loss > 500 mL<br>3. evaluation of the number of cases with global blood loss > 1000 mL<br>4. evaluation of the number of cases with global blood loss > 2000 mL<br><br>xxxxxxxxxxxxxxxxxxxxxxxxxxxxxxxxxxxxxxxxxxxx

**Secondary outcomes**	1. evaluation of the number of cases in which the additional use of Oxytocin, Sulprostone, Misoprostol, Methylergometrina, tranexamic adic, VII factor was necessary<br>2. evaluation of the number of cases in which the additional use of surgical maneuvers for bleeding control (need of intrauterine balloon or uterine compression sutures for surgical treatment of the PPH or embolization or hysterectomy has been necessary<br>3. evaluation of the number of cases in which hemodynamic changes occur: hypotension (number of women with PA < 100/60 mm/Hg) and increased heart rate (number of women with FC > 60 bpm)<br>4. evaluation of the number of cases with Hb <7g / dl on the second day after delivery<br>5. evaluation of the number of cases in which blood transfusions were necessary<br>6. evaluation of the number of cases in which the following was seen verified:<br>• nausea between delivery and discharge<br>• vomiting between birth and discharge<br>• headache between birth and discharge<br>• dyspnoea between birth and discharge<br>• Chest pain between birth and discharge<br>• endometritis after delivery (assessed by monitoring body temperature)
**Safety outcomes**	xxxxxxxxxxxxxxxxxxxxxxxxxxxxxxxxxxxxxxxx<br>1) numbers of cases of DVT(visit V6 et V7) Objective evaluation considering the following suggestive factors (in addition to those evaluated at the vistit V0 and V1):<br>post partum hemorrhage ≥ a 1000 ml with or without surgery,transfusions,inflammatory process and post partum infections,immobility > 4 days before delivery or after delivery,dehydratation<br>2) numbers of cases of seizures (visits from V2 to V7):<br>Objective evaluation: beginning, frequency,

	conditions at the time of crisis (es. concomitance of fever)<br><br>3) numbers of cases of visual disorders (= alterated color perception) (visits from V2 to V7): numbers of events , date of emergence from the assumption of the study drugs, regression time, persistence of semi-darkness<br><br>4) numbers of serious and non-serious adverse drug reactions, not reported in the Summary of the product (visits from V2 to V7)
**Inclusion Criteria**	1. Patients at the end of pregnancy (37-42 w) at low risk of PPH, age major or equal to 18 years old, mean by low-risk patients without any of the following risk factors:<br>- Hypertension<br>- Preeclampsia<br>- placental abruption during pregnancy<br>- placental previa<br>- tocolysis two hours before giving birth<br>- multiple pregnancy<br>- previous PPH<br>- obesity (BMI > 35)<br>- anemia (Hb < 7 g/dL)<br>- elective caesarean section<br>- induction of labor<br>- retention of placental material polyhydramnios<br>- fever during labor<br>- use of heparin low molecular weight<br>2. Signing the informed consent
**Exclusion criteria**	1. Patients with pre-term pregnancy (<37 weeks)<br>2. Patients with prolonged pregnancy (> 42 weeks)<br>3. Patients at the end of pregnancy (37 weeks - 42 weeks) with the following risk factors for PPH (reported in Table 1)<br>4. Patients with Long - QT syndrome or who are taking drugs that cause QT prolongation<br>5. intrauterine fetal death<br>6.kidney deficiency<br>7.epilepsy<br>8.autoimmune disease<br>9. history of thromboembolic disorders or High incidence of thromboembolic events in family history (Patients at high risk thrombophilia)

	10) patients minor of 18 years old<br>11) time between V0 and V1 less than 1 week
**Dropouts**	• Caesarean sections in labour: the expected 11%<br>• Patients who used OXY in labour: the expected 10%<br>• Vaginal operative deliveries: the expected 7.5%<br>• The suspension or therapeutic changes due to adverse events<br>• Withdrawal of the patient<br><br>The reasons of the output from the study will be documented in the CRF (data collection form). Patients should be informed about their right to withdraw from the study at any time.

Statistical analysis

For the primary outcome the assumption of equivalence will be verified by two tests: 1) Westlake - Schuirmann equivalence test. For each of the two following tests of non-inferiority, a significant result rejects the hypothesis indicated:

- hypothesis 1: "A" exceeds "B" of 150 ml or more
- hypothesis 2: "B" exceeds "A" of 150 ml or more

A and B are equivalent if both tests are significant (two-tail $p<0.05$).

Confidence interval of 95% of the observed difference between A and B. If the confidence interval equal to 95% falls completely within the range of -150 to 150 will reject the null hypothesis that the difference exceeds 150, and is supported equivalence between the two treatments (p two tails <0.05).

For secondary outcomes the comparison between treatments will be made through the Fisher's exact test to check if there are significant differences.

Safety

During the study we will collect all adverse events/adverse reactions occurred during the experimentation [for the definition of adverse events, serious and not, please refer to the standards of Good Clinical Practice of the International Conference on Harmonisation (incorporated in the Protocol), in the manner and time required by applicable law (D.Lvo 211/2003 - DM 21/12/2007 - Good Clinical Practice (GCP-ICH), AIFA 20/09/2012). All adverse events will be collected through the CIOMS form/AE form, recorded on the patient's medical record and on CRF. The investigator will assess the severity, and causal association with IMP with regards to any adverse event recorded on the CRF. The investigator will assess: event severity, causal relationship between IMP and/or concurrent therapy and the adverse event, if the event is expected or not expected compared with the reference document referred to in Protocol (RCP product). Each Suspected Adverse Reaction related to an IMP (drug under study and comparator drug) and that is both Unexpected Serious (SUSAR), will be forwarded to the Eudravigilance database, as well as the CE competent authority which issued the approval.
