# Supplementary material for: Tranexamic acid versus oxytocin prophylaxis in reducing post-partum blood loss, in low-risk pregnant women: TRANOXY STUDY, a phase III randomized clinical trial
Source: eClinicalMedicine. 2024 May 31;73:102665. doi: 10.1016/j.eclinm.2024.102665 (PMC11169955; doi:10.1016/j.eclinm.2024.102665)
Supplement: Appendix [file mmc2.docx]

**Tranexamic acid versus oxytocin prophylaxis in reducing post-partum blood loss, in low-risk pregnant women: TRANOXY STUDY, a phase III randomized clinical trial.**

Antonio Ragusa et al.

**SUPPLEMENTARY APPENDIX**

Table of Contents

List of Steering Committee members............................................................................................................ 3 List of Endpoint Committee members.......................................................................................................... 4 Data Safety and Monitoring Board................................................................................................................ 5 In- and exclusion criteria............................................................................................................................... 6 List of standardized procedures performed at time of admission of the patients........................................ 8 Authorisation of the ethics committee.......................................................................................................... 9 Statistical considerations…………………………………………………..…………………………………………………………………….10 Supplementary Tables…………………………………………………………………………………………………………………………….11 Supplementary Figures.................................................................................................................................14

| **List of Steering Committee members**  Antonio Ragusa^a^, Fernando Ficarola^b,c*^, Amerigo Ferrari^d^, Nicoletta Spirito^e^, Mario Ardovino^f^, Domenico Giraldi^f^, Elisario Stuzziero^f^, Denise Rinaldo^g^, Roberto Procaccianti^h^, Giovanni Larciprete^i^, Caterina De Luca^i^, Sara D’Avino^i^, Giulia Principi^j^, Roberto Angioli^b^, Alessandro Svelato^i^  ^a^ Department of Obstetrics and Gynecology, Maggiore Hospital Carlo Alberto Pizzardi Bologna, Italy  ^b^ Unit of Gynecology, Fondazione Policlinico Universitario Campus Bio-Medico, Rome, Italy  ^c^ Unit of Gynecology, Department of Surgical and Medical Sciences and Translational Medicine, Sant'Andrea Hospital, Sapienza University of Rome, Rome, Italy.  ^d^ Institute of Management, MeS (Management and Health) Laboratory, Sant’Anna School of Advanced Studies, Pisa, Italy  ^e^ Department of Obstetrics and Gynecology, Ospedale Apuane, Massa Carrara, Italy  ^f^ Department of Obstetrics and Gynecology Ospedale S.G. Moscati, Avellino  ^g^ Department of Obstetrics and Gynecology, ASST Bergamo Est, Bolognini Hospital, Seriate, Bergamo, Italy  ^h^ Department of Gynecology and Obstetrics, Fondazione Istituto San Raffaele G Giglio, Cefalù, Italy  ^i^ Department of Obstetrics and Gynecology, Fatebenefratelli Gemelli Hospital, Isola Tiberina, Roma, Italy  ^j^ Department of Obstetrics and Gynecology, University of Messina, Messina, Italy  **List of Endpoint Committee members**  The members of the endpoint committee consist of experts in Obstetrics and management of postpartum hemorrhage in vaginal birth.  Antonio Ragusa: Department of Obstetrics and Gynecology, Maggiore Hospital Carlo Alberto Pizzardi Bologna, Italy  Elisario Stuzziero: M.D., Chief of Department of Obstetrics and Gynecology Ospedale S.G. Moscati, Avellino.  Roberto Angioli: PhD, Unit of Gynecology, Fondazione Policlinico Universitario Campus Bio-Medico, Rome, Italy  Alessandro Svelato: M.D., Department of Obstetrics and Gynecology, Fatebenefratelli Gemelli Hospital, Isola Tiberina, Roma, Italy.  **Data Safety and Monitoring Board**  Members:  Chair: Antonio Ragusa  Address: Department of Obstetrics and Gynecology, Maggiore Hospital Carlo Alberto Pizzardi Bologna, Italy  Email: Antonio.ragusa@gmail.com  Telephone: +393355726903  Discipline: Gynecology and Obstetrics  Name: Elisario Stuzziero  Address: Department of Obstetrics and Gynecology Ospedale S.G. Moscati, Avellino  Email: strudoc@gmail.com  Telephone: +393357886944  Discipline: Obstetrics and Gynecology  Name: Alessandro Svelato  Address: Department of Obstetrics and Gynecology, Fatebenefratelli Gemelli Hospital, Isola Tiberina, Via di Ponte Quattro Capi, 39, 00186 Roma, Italy.  Email: alessandrosvelato@virgilio.it  Telephone: +393491272580  Discipline: Obstetrics and Gynecology  **In- and exclusion criteria**  Inclusion Criteria: The enrolled pregnancy population consists of full-term patients (37-42 weeks) at low risk of PPH.  Exclusion Criteria:  List of risk factors (the presence of even one of these factors excluded the possibility of participating in the study)   1. hypertension; 2. preeclampsia; 3. placental abruption in pregnancy; 4. placenta previa; 5. tocolysis two hours before childbirth; 6. twinning; 7. previous PPH; 8. obesity (BMI> 35); 9. anaemia (Hb< 7 g/dL); 10. elective caesarean section; 11. induction of childbirth; 12. polyhydramnios; 13. fever in labor; 14. use of low molecular weight heparin. 15. patients with preterm pregnancy (<37 W); 16. patients with prolonged pregnancy (>42 W); 17. patients with QT-Long syndrome or taking medications that can cause QT stretching; 18. MEF (intrauterine fetal death); 19. Renal; 20. epilepsy; 21. autoimmune diseases; 22. presence in remote pathological anamnesis or in family history of thromboembolic events.   **List of standardized procedures performed at time of admission of the patients**   1. detailed collection of medical history aimed at recording information on medical, surgical, obstetric history and any pharmacological therapies in place; 2. verification of eligibility criteria; 3. delivery of the information sheet and consent after having explained the study to the patient and clarified doubts and perplexities, and having received the patient’s acceptance; 4. collection of informed consent signed for participation in the study; 5. recording of weight, height, heart rate, blood pressure and temperature of the patient in the folder; 6. execution of blood sampling for blood chemistry tests (blood count, PT, PTT, fibrinogen).   Near delivery, the eligibility to participate in the study was re-evaluated and, if confirmed, one of the two treatment groups provided for in the study protocol was randomized by the midwife, who opened a sealed envelope.  Immediately after delivery, as per clinical practice already in use, blood loss was monitored by means of a graduated bag placed under the patient’s pelvis, which was removed only after expulsion of the placenta and completion of any vagino-perineal suture, after evaluation and recording of the blood loss contained in it.  In both groups, cord clamping was carried out after 60 seconds from birth, and administration of the drug was performed within 5 minutes. Controlled traction of the umbilical cord was never performed.  At 24 hours after delivery, blood chemistry (blood count, PT, PTT, fibrinogen) and detection of maternal parameters was performed.  40 days after delivery, a telephone interview was scheduled to assess the patient’s clinical health conditions, with particular attention to the possible onset of thromboembolic diseases .  Patients were informed of the possibility of leaving the study at any time, without any change in clinical management. The reasons for abandoning the study were documented in the data collection card if this occurred. Patients who left the study, in the opinion of the investigator, due to discontinuation or therapeutic modification due to adverse events, were treated according to usual management.  **Authorisation of the ethics committee**  *“Comitato Etico Regionale per la Sperimentazione Clinica della Regione Toscana. Sezione: AREA VASTA NORD OVEST, ubicato c/o: Stabilimento di Santa Chiara - Via Roma, 67 -56126 Pisa. Telefono: 050/996247-2757. E-mail:staffamm.ce@ao-pisa.toscana.it PARERE PRESA D'ATTO nella seduta del 06/12/2018. Numero registro pareri del Comitato Etico:* ***2016-1129. Prot n 63209”*.** The trial was performed in accordance with the principles of the Declaration of Helsinki. The authors assume responsibility for the accuracy and completeness of the data and analyses, as well as for the fidelity of the trial and this report to the protocol.  **Statistical considerations**  The primary objective of our study was to evaluate the equivalence of the two treatment groups in the management of PPH.  For this purpose, the T test or, when appropriate, the nonparametric Mann-Whitney test, was applied to evaluate the difference in continuous variables between the two groups (OXY and TRAN).  For the main outcome, the equivalence hypothesis was tested by using the equivalence test called 'two one-sided t-tests' (TOST) procedure. For each of the following two one-tail non-inferiority tests, a significant result rejects the hypothesis given:  -Hypothesis 1: A exceeds B by 150ml or more  -Hypothesis 2: B exceeds A by 150ml or more  A and B are equivalent if both tests are significant (two-tailed p <0.05).  We also computed the 95% confidence interval of the observed difference between A and B. If the 95% confidence interval fell completely within the -150 to 150 range, the null hypothesis that the difference exceeds 150 was rejected, and equivalence between the two treatments was supported (two-tailed p <0.05).  Since the equivalence test was repeated for T0 blood loss and total blood loss, the p-values were corrected through the Benjamini-Hochberg procedure for false discovery rate (FDR) control.  Continuous data were represented in terms of median (interquartile range, IQR), while categorical data were represented in terms of absolute frequency and percentage (%).  All analyses were performed on Stata Software version 17.0 (Stata-Corp, LLC, College Station, Texas, USA).  **Supplementary Tables**   \| Outcomes \| **OXY** \|  \| **TRAN** \|  \| p-value \| \| --- \| --- \| --- \| --- \| --- \| --- \| \|  \| (n=127) \|  \| (n=104) \|  \| \| T0 blood loss, median (IQR) \| 150.0 (100.0, 300.0) \| \| 200.0 (100.0, 350.0) \| \| 0.86 \| \| T0 blood loss, n (%) \|  \|  \|  \|  \| 0.82 \| \| ≤1000 mL \| 124 (97.6%) \| \| 102 (98.1%) \| \|  \| \| >1000 mL \| 3 (2.4%) \| \| 2 (1.9%) \| \|  \| \| 2-hour blood loss (lochia), median (IQR) \| 50.0 (30.0, 93.0) \| \| 80.0 (30.0, 104.0) \| \| 0.13 \| \| Total blood loss, median (IQR) \| 260.0 (170.0, 477.0) \| \| 323.0 (180.0, 555.0) \| \| 0.46 \| \| Total blood loss, n (%) \|  \|  \|  \|  \| 0.97 \| \| ≤1000 mL \| 106 (83.5%) \| \| 87 (83.7%) \| \|  \| \| >1000 mL \| 21 (16.5%) \| \| 17 (16.3%) \| \|  \| \| Hb 24h, median (IQR) \| 10.5 (9.8, 11.5) \| \| 10.7 (9.8, 11.5) \| \| 0.78 \|   **Supplementary Table S1. Outcomes.**  We performed the Mann-Whitney U-test for continuous variables and the chi-square test for categorical variables to show that there was no statistically significant difference between the two study groups in any study outcome.   \|  \| \| \| \| \| \| --- \| --- \| --- \| --- \| --- \| \|  \| Total blood loss >1000mL \| \|  \| **TOST** \| \|  \| Mean \| 95% CI \|  \| \| Oxytocin (n=127) \| 0,17 \| 0,10 \|  \| *Test for difference* \| \| Tranexamic acid (n=104) \| 0,16 \| 0,09 \|  \| H0: no difference (diff = 0) \| \|  \|  \|  \|  \| p-value > 0.05 (not rejected) \| \| Difference \| 0,00 \| -0,09 \|  \|  \| \|  \|  \|  \|  \| *Test for equivalence* \| \|  \| (Sample proportions) \| \|  \| H0: no equivalence (\|θ\| ≥ Δ) \| \|  \|  \|  \|  \| p-value > 0.05 (not rejected) \| \|  \|  \| \|  \|  \| \|  \|  \|  \|  \|  \| \| Equivalence limits (Δ) \| 0.03 (3%) \| \|  \| Conclusion: **Indeterminate** (underpowered test) \| \|  \|  \|  \|  \|  \| \|  \|  \|  \|  \|  \| \|  \|  \|  \|  \|  \| \|  \| Hemoglobin at 24 hours \| \|  \| **TOST** \| \|  \| Mean \| 95% CI \|  \| \| Oxytocin (n=115) \| 10,62 \| 10,37 to 10,87 \|  \| *Test for difference* \| \| Tranexamic acid (n=100) \| 10,62 \| 10,35 to 10,89 \|  \| H0: no difference (diff = 0) \| \|  \|  \|  \|  \| p-value > 0.05 (not rejected) \| \| Overall (n=215) \| 10,62 \| 10,44 to 10,8 \|  \|  \| \|  \|  \|  \|  \| *Test for equivalence* \| \| Difference \| 0,00 \| -0,367 to 0,36 \|  \| H0: no equivalence (\|θ\| ≥ Δ) \| \|  \|  \|  \|  \| p-value < 0.05 (rejected) \| \| p-value for normality \| >0.05 (normal) \| \|  \|  \| \|  \|  \|  \|  \|  \| \| Equivalence limits (Δ) \| 0.4 g/dL \| \|  \| Conclusion: **equivalence** \| \|  \|  \|  \|  \|  \| \| **Supplementary Table S2.** **TOST.** We performed the TOST procedure for the secondary outcomes 2A) Total blood loss >1000mL, and 2B) Hemoglobin at 24 hours. We found that the proportion of total blood loss >1000mL did not significantly differ between the study groups, but at the same time the test was underpowered to draw conclusions concerning equivalence. On the contrary, we did find equivalence in Hemoglobin levels between the study groups within an equivalence margin of 0.4 g/dL of Hemoglobin  procedure for the secondary outcomes \| \|  \|   We performed the TOST procedure for the secondary outcomes 2A) Total blood loss >1000mL, and 2B) Hemoglobin at 24 hours. We found that the proportion of total blood loss >1000mL did not significantly differ between the study groups, but at the same time the test was underpowered to draw conclusions concerning equivalence. On the contrary, we did find equivalence in Hemoglobin levels between the study groups within an equivalence margin of 0.4 g/dL of Hemoglobin   \|  \| \| \| \| \| --- \| --- \| --- \| --- \| \| **Adverse reactions** \| **OXY**  (n=127) \| **TRAN**  (n=104) \| p-value * \| \| Nausea \|  \|  \| 0.90 \| \| No \| 122 (96.8%) \| 101 (97.1%) \|  \| \| Yes \| 4 (3.2%) \| 3 (2.9%) \|  \| \| Vomiting \|  \|  \| 0.22 \| \| No \| 122 (96.1%) \| 96 (92.3%) \|  \| \| Yes \| 5 (3.9%) \| 8 (7.7%) \|  \| \| Diarrhea \|  \|  \| 0.18 \| \| No \| 124 (97.6%) \| 98 (94.2%) \|  \| \| Yes \| 3 (2.4%) \| 6 (5.8%) \|  \| \| Chills \|  \|  \|  \| \| No \| 127 (100.0%) \| 104 (100.0%) \|  \| \| Headache \|  \|  \| 0.097 \| \| No \| 121 (95.3%) \| 103 (99.0%) \|  \| \| Yes \| 6 (4.7%) \| 1 (1.0%) \|  \| \| Allergic reactions \|  \|  \| − \| \| No \| 127 (100.0%) \| 104 (100.0%) \|  \| \| Thromboembolic reactions \|  \|  \| − \| \| No \| 127 (100.0%) \| 104 (100.0%) \|  \| \| Intensive care unit (mother) \|  \|  \| − \| \| No \| 127 (100.0%) \| 104 (100.0%) \|  \| \| Death \|  \|  \| − \| \| No \| 127 (100.0%) \| 104 (100.0%) \|  \| \| * chi-square test p-value \| \| \| \|   **Supplementary Table S3. Adverse reactions (safety outcomes)**  **Supplementary Figures**  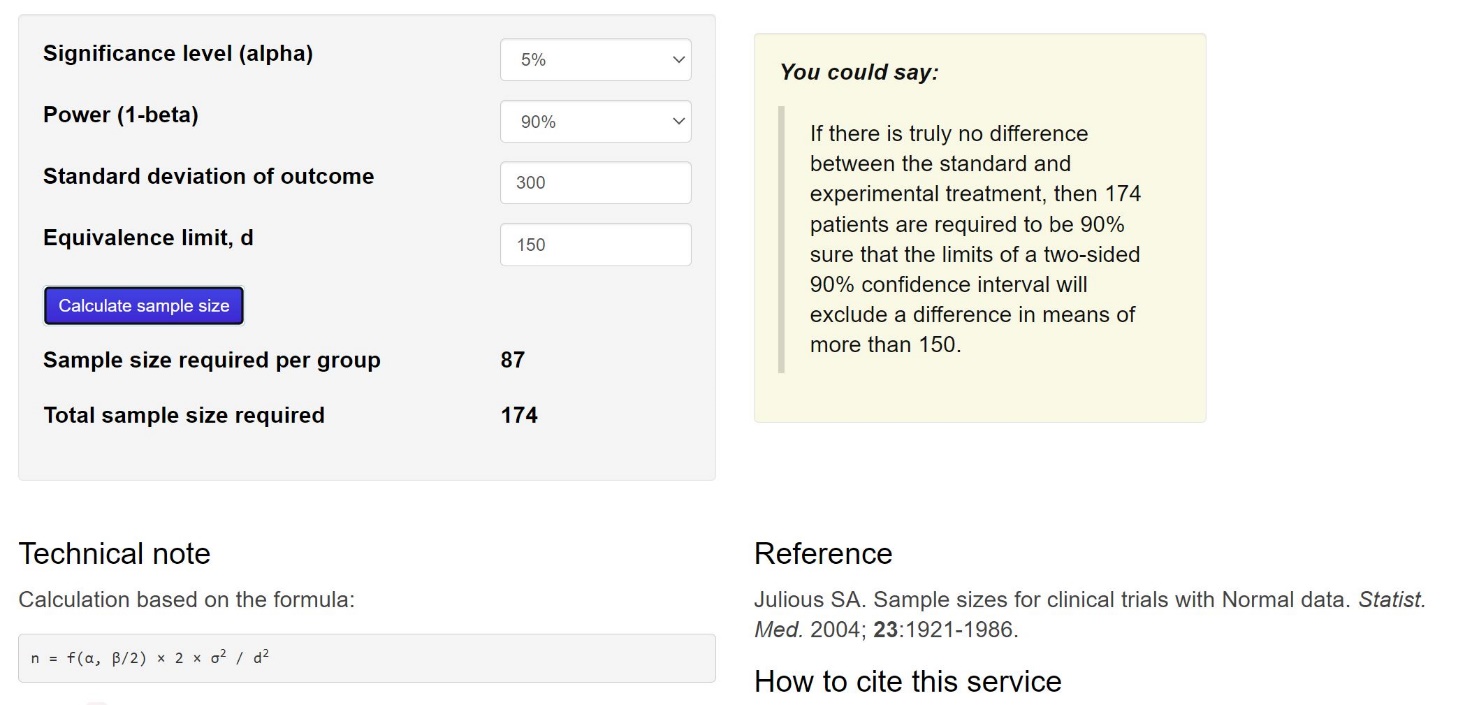  **Supplementary Figure 1. Sample size calculation** |  |  |  |  |  |
| --- | --- | --- | --- | --- | --- | --- | --- | --- | --- | --- | --- | --- | --- | --- | --- | --- | --- | --- | --- | --- | --- | --- | --- | --- | --- | --- | --- | --- | --- | --- | --- | --- | --- | --- | --- | --- | --- | --- | --- | --- | --- | --- | --- | --- | --- | --- | --- | --- | --- | --- | --- | --- | --- | --- | --- | --- | --- | --- | --- | --- | --- | --- | --- | --- | --- | --- | --- | --- | --- | --- | --- | --- | --- | --- | --- | --- | --- | --- | --- | --- | --- | --- | --- | --- | --- | --- | --- | --- | --- | --- | --- | --- | --- | --- | --- | --- | --- | --- | --- | --- | --- | --- | --- | --- | --- | --- | --- | --- | --- | --- | --- | --- | --- | --- | --- | --- | --- | --- | --- | --- | --- | --- | --- | --- | --- | --- | --- | --- | --- | --- | --- | --- | --- | --- | --- | --- | --- | --- | --- | --- | --- | --- | --- | --- | --- | --- | --- | --- | --- | --- | --- | --- | --- | --- | --- | --- | --- | --- | --- | --- | --- | --- | --- | --- | --- | --- | --- | --- | --- | --- | --- | --- | --- | --- | --- | --- | --- | --- | --- | --- | --- | --- | --- | --- | --- | --- | --- | --- | --- | --- | --- | --- | --- | --- | --- | --- | --- | --- | --- | --- | --- | --- | --- | --- | --- | --- | --- | --- | --- | --- | --- | --- | --- | --- | --- | --- | --- | --- | --- | --- | --- | --- | --- | --- | --- | --- | --- | --- | --- | --- | --- | --- | --- | --- | --- | --- | --- | --- | --- | --- | --- | --- | --- | --- | --- | --- | --- | --- | --- | --- | --- | --- | --- | --- | --- | --- | --- | --- | --- | --- | --- | --- | --- | --- | --- | --- | --- | --- | --- | --- | --- | --- | --- | --- | --- | --- | --- | --- | --- | --- | --- | --- | --- | --- | --- | --- | --- | --- | --- | --- | --- | --- | --- | --- | --- | --- | --- | --- | --- | --- | --- | --- | --- | --- | --- | --- | --- | --- | --- | --- | --- | --- | --- | --- | --- | --- | --- | --- | --- | --- | --- | --- |
